# Strategic concept paper for Bioeconomy in Slovenia: from a patchwork of good practices to an integrated, sustainable and robust bioeconomy system

**DOI:** 10.12688/openreseurope.16181.1

**Published:** 2023-10-03

**Authors:** Luka Juvančič, Sabina Berne, Primož Oven, Ilja Gasan Osojnik Črnivec

**Affiliations:** 1 Biotechnical Faculty, University of Ljubljana, Ljubljana, Ljubljana, 1000, Slovenia

**Keywords:** Slovenia, bioeconomy, value chains, biomass streams, industrial innovation, logistics, firm consolidation, strategic planning

## Abstract

While Slovenia has significant bioeconomy potential, it remains underutilized, facing challenges in primary bioeconomy sectors, their integration along value chains, uptake of industrial innovation, and institutional coordination. This paper aims to support the unlocking of Slovenia's bioeconomy potential, and foster sustainable and integrated development of its value chains. It provides the evidence base of the composition, volumes and current utilization of the available biomass streams from agriculture, forestry and aquatic systems. It discusses the potential uses of these resources and highlights the need for improved logistics and scalability. Additionally, the structure and performance of bioeconomy-related industries in Slovenia are examined, emphasizing the importance of firm consolidation and integration for successful bioeconomy development. It emphasizes the importance of sector-specific transformation pathways, from primary production to expanding hybrid sectors. The exchange between policymakers and stakeholders is encouraged to recognize synergies, accelerate cooperation, and improve economic performance while closing material and energy loops. The document also reviews the supporting environment for bioeconomy development and proposes steps for improved coordination and strategic planning.

## Disclaimer

The views expressed in this article are those of the author(s). Publication in Open Research Europe does not imply endorsement of the European Commission.

## Plain language summary

Slovenia has great potential for using its natural resources in a more sustainable way, but it is not fully taking advantage of this opportunity. There are several challenges that need to be addressed, such as integrating different sectors of the economy, promoting innovation in industries, and improving coordination between institutions. This analysis provides information about the types of natural resources available, such as biomass from agriculture, forestry, and aquatic systems, and how they are currently being used in Slovenia. The paper also examines the industries related to the bioeconomy in Slovenia and highlights the importance of companies working together and integrating their efforts for successful development. The paper discusses the different ways these resources can be used and emphasizes the way to unlock the bioeconomy potential of Slovenia and promote the development of sustainable and integrated value chains. To achieve these goals, it is important for policymakers and stakeholders to communicate and work together. This will help identify opportunities for collaboration, accelerate progress and strengthen the bioeconomy sectors.

## Introduction

This concept paper is an output of the BIOEASTsUP project, whose overall objective is to support Slovenia (and other countries participating in the BIOEAST initiative) in the unlocking of its bioeconomy potentials. While it largely builds on the findings of the related project deliverables ( Juvančič *et al.*, 2021a; Kulišić *et al.*, 2020; Vitunskienė *et al.*, 2021), it also takes into account some previous research and strategic planning effort with a similar focus. In this context, we highlight the nationally funded project BRIDGE2BIO ( Juvančič *et al.*, 2021b), BBI JU CSA project CELEBio ( Virant *et al.*, 2020) and some national strategic documents, most notably the Comprehensive Strategic Project of Decarbonisation ( Karba, 2022). These documents provide a solid foundation for creating the bioeconomy strategy by defining sector-specific transformation pathways towards unlocking the potentials for a more sustainable, integrated and better performing bioeconomy in Slovenia.

The concept paper aims to provide the evidence base and improve the flow of information between the policymakers and stakeholders. It is meant to contribute towards aligning different views about relevant pathways of bioeconomy sectors in Slovenia—from primary production (agriculture, forestry, aquatic production systems) and conventional bioeconomy manufacturing sectors (food products and beverages, wood processing, pulp and paper), to the expanding ‘hybrid’ bioeconomy sectors (manufacture of chemical products, textiles, construction, pharmaceutical preparations), as well as energy supply, and service sectors engaged in ecosystem services valorisation. Ideally, this exchange should prove beneficial for enterprises and other economic entities operating in various bioeconomy sectors in Slovenia, to recognise their synergies and accelerate cooperation in integrated value chains. The projected improvements are not meant only in terms of the improved economic performance of participating companies, but also in terms of the aggregate gains of total value added created along (extended) biobased value chains, as well as improved sustainability of the economic system by closing (material, energy) loops of biomass utilisation.

The aim of the concept paper is also to review and critically assess the supporting environment for the development of the bioeconomy in Slovenia. Dedicated strategic framework and coordinated policy support can initiate actions that improve the overall economic performance, as well as accelerate the processes of the restructuring of bioeconomy in the direction of resilience and sustainability of the economic system. The concept paper is developing some proposals in this regard. Rather than suggesting a developed set of instruments and measures, the policy recommendations are meant to discuss the status of the bioeconomy in the current institutional setup and system of development planning in Slovenia. They are meant to provide a basis for informed coordination among policy makers in planning future actions to support the sustainable and integrated development of the national bioeconomy.

## Current state of the system components, opportunities and challenges

### Agricultural production and residual biomass from the agri-food chain

Natural limitations (58% of utilised agricultural area is dominated by grassland, three quarters of agricultural land is located in areas with natural and other restrictions) determine the scope and structure of primary agricultural production. Accordingly, two-thirds of agricultural holdings are engaged in livestock production, where (increasingly specialized) cattle breeding for meat and milk production dominate. Animal production, together with own feed production, contributes the largest share (56%) to the value of agricultural production. There are untapped reserves in the integration of primary production to the local food chains, as aforementioned branches of agriculture, almost a third of the total production is exported as a basic raw material (raw milk or live animals). On the other hand, even in the sectors facing steep growth in demand ( *e.g.*, fresh vegetables, organic food), the supply-side is struggling to establish systems suitable that would be able to supply the most frequent retail formats. This observation can be extended to the entire agri-food sector in Slovenia: weak vertical integration along the food value chain, is a key obstacle in the operation of the Slovenian food system.

Among the residual streams of agricultural biomass, by far the largest quantity is attributed to livestock excrements with a total amount of more than 620 thousand tons of dry matter. The overall performance of its current use (organic fertilizer) can be significantly improved by exploiting its energy content (biogas production) prior to the fertilization, and by improving soil fertilization techniques, which improves the nutrient utilization and reduces environmental burden.

When selecting raw materials and preparing a technological design for the circular use of residues and by-products of plant production, we proceed from two principles. First, that the proposed solutions should not threaten the balance of organic matter in the soil. Secondly, they need to take into account the structural features of farming in Slovenia (small-scale and fragmented property structure). The most extensive raw material source in plant production is represented by harvest residues and secondary crops of arable production, the total amount is in the range of 700,000 tons of dry matter. The remains of vegetable, oil and root crops represent the next quantitatively and qualitatively perspective raw material source, the total amount is in the range of 100,000 tons of dry matter. Other potentially relevant raw material source, are also residues in horticulture, amounting to 30,000 tons of dry matter.

When searching for alternatives for circular use of above listed perspective groups of agricultural biomass, we must take into account either their limitations in ensuring efficient logistics and scalability, and ecologic limitations. However, these biomass streams provide the potentials for technologically and economically sound circular uses, such as: (i) cascading use of lignocellulosic residues with an emphasis on the extraction of bioactive components and the production of packaging materials; (ii) transformation of biomass with a high fibre content into composite materials or (iii) biorefining of more complex raw material sources ( *e.g.*, residues from the processing of fruits, vegetables and oilseeds into components with a high added value).

Considering the chemical composition and technological properties of side streams in food processing, there are untapped potentials in the extraction of bioactive compounds and application of various biotechnological processes. The range of compounds obtained is extensive and offers a strong potential for adding value. Our research identified unexploited reserves particularly in the sectors, which provide homogenous streams of biomass and allow for scalability. Such sectors are dairy, animal by-products, brewing industry and wine production.

### Availability and possible uses of Forest-wood biomass

With an exceptional forest cover (58% of the country area are forests with a relatively strong production capacity), wood is by far the most promising source of raw materials in the Slovenian bioeconomy. This potential is somewhat limited by a fragmented ownership structure (average size of a forest property is 2.9 ha), which is the main drawback for organizing cost-efficient supply of wood biomass at the industrial scale. Furthermore, the structure and production potential of Slovenian forests is irreversibly changing due to climate change. Future projections forecast an increase of hardwood potential, particularly from the increasing share and faster growth of the beech forests.

The average yearly production of forest wood assortments in Slovenia amounts to about 4.5 million m ^3^, about two thirds of these are conifers. The largest domestic consumer of round wood is the sawn wood industry (over 1 million m ^3^), followed by the wood composites, mechanical pulp and chemical industries with a total processing volume of around 0.5 million m ^3^. Large consumers of round wood are households, which annually consume over 1 million m ^3^ of wood for firewood. Slovenia is a prominent exporter of unprocessed round wood, which is particularly evident in the coniferous log category with about 1.3 million m ^3^.

Looking from the viewpoint of the overall economic performance of the forest-wood related bioeconomy in Slovenia, the current situation is not favourable. Improvements are sought in particular in terms of a higher share of harvested round wood processed domestically, and in the strengthening of more technologically advanced alternatives to the current uses of round wood. Reserves exist also in the enhanced exploitation of the economic potential of the forest, as currently, only 60-70% of the annual increment of wood is harvested. The largest potentials are estimated for the wood categories of lower quality. From the point of view of the long-term perspective, this category will gain in importance with changes in forest stands (increasing proportion of beech). Unexploited possibilities are therefore especially in the categories of wood, which are a suitable input raw material for biorefining processes and the subsequent production of new bio-based materials.

The potential of logging residues for collection and processing in industrially relevant quantities is limited, as their removal is not cost-efficient. Some bioeconomic potential in this category can be attributed to bark, which by volume represents around 20% of the cut and is an important category of raw materials for bio-based products due to a high content of bioactive compounds ( *e.g.*, tannins, polyphenols) and is also a good structural material for composting biogenic waste.

### Structure and performance of bioeconomy – related manufacturing sectors

The experience of the leading EU countries and regions reveals that sectors with strong, consolidated firms in conventional bioeconomy sectors find it easier to provide leverage for the development of industrial-scale biorefineries and the resulting potentials for value-adding ( European Commission, 2022; Gardossi *et al.*, 2023). Slovenia has a vibrant structure of enterprises engaged in conventional bioeconomy-related industries (food processing, wood processing, paper mills), but most of these operate at the small and medium-sized enterprise (SME) scale. Conventional bioeconomy manufacturing sectors are relatively strongly represented on international markets. Enterprises operating in wood processing achieve 55% of revenues on international markets, whereas the corresponding share for the food processing sector is 34%, which is below the par of the manufacturing sector in Slovenia ( Juvančič *et al.*, 2021b).

The scale and the level of integration of industrial operations in these sectors significantly dropped throughout the political transition and economic restructuring in the 1990s ( Polanec, 2007). Some industrially-relevant operations that could serve as the core for future industrial-scale biorefineries, ceased with their operations in the last two decades. The level of business integration in conventional bioeconomy-related industries is rather low (vertically, as well as horizontally), which prevents the scale effects needed for a functioning of the 'enhanced' bioeconomy concept, integrating firms in the same, or complimentary sectors, with a biorefinery at its core. In the development of more diversified and innovative bio-based value chains, two scenarios seem feasible: (i) integration into bioeconomic clusters, with a network of small-scale modular biorefinery operations in its core, or (ii) integration into wider, cross-border value chains, supplying biomass to, and supplying intermediate outputs from industrial biorefineries, located within operating distance from Slovenia.

Apart from the ‘conventional’ bioeconomy sectors, integration of firms operating in technology-intensive sectors that are strongly integrated into international value chains ( *e.g.*, chemical industry, automotive sector) may also play a catalytic role in the transition towards bioeconomy. Demand for biobased technologies and components in these industries is increasing at an accelerated pace. A number of factors, such as disruptions on global raw material markets, technological prowess in biobased technologies and changed price-cost relationships, are simultaneously contributing towards the accelerated turn towards innovative biobased technologies in sectors that were traditionally operating with non-renewables. Increased demand for biobased technologies and components in technology-intensive sectors may serve as an important engine of growth also in ‘conventional’ biobased sectors ( Lovec & Juvančič, 2021). Apart from being the providers of biomass (often with poorly-valorised side-streams), integration with technology-intensive sectors may act as a stimulus to improve their performance in several aspects (closing the material and energy loops, improved economic performance).

### Economic performance of the bioeconomy sectors in Slovenia through the lens of labour productivity

Value added per employee as a measure of labour productivity in the bioeconomy in Slovenia in 2017 reached 20,519 EUR. The highest labour productivity is recorded in the (hybrid) sector of pharmaceutical manufacturing, where the added value per employee is above 135,000 EUR. This is followed by electricity production with almost 90,000 EUR, chemical industry with 64,200 EUR and beverage production with 55,800 EUR. Industries with labour productivity below 30,000 EUR are food production, wood processing, leather production, fishing and clothing production (figures ranging between 18,150 EUR and 28,600 EUR). Agriculture records the lowest added value per employee, 7,130 EUR.

**Figure 1.**
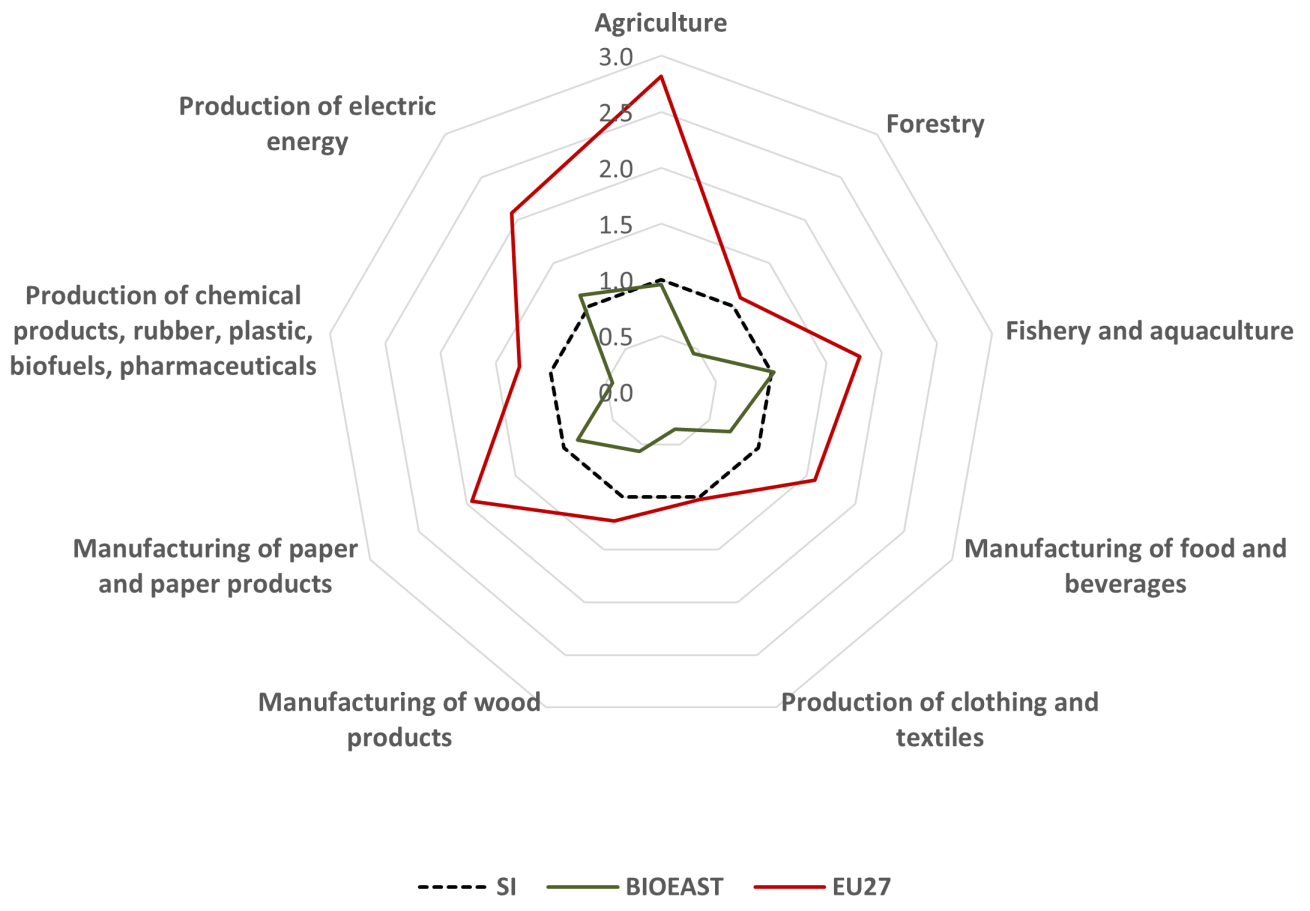
Comparison of labour productivity (VA/employee) by bioeconomy sectors in Slovenia, EU27 and BIOEAST. Source of data: Ronzon *et al.*, 2018.

In general, labour productivity in bioeconomy in Slovenia is relatively low. It is almost as twice as large as in the BIOEAST region (11,500 EUR) but lags far behind the EU27 average of 35,000 EUR. Compared to the EU27, labour productivity is particularly low in agriculture, in electricity production and in the paper industry ( Figure 1).

An insight into the temporal component reveals additional structural features of the bioeconomy sectors. The highest growth in added value per employee during the period 2008–2017 was recorded in the production of textiles and clothing, but mainly on the account of an intense decline in the number of employees (-60%). Similar, albeit in a less pronounced manner applies to the wood processing industry and paper production. Production of aquatic organisms records the highest labour productivity growth (89%), which is clearly the result of an equally intense increase in added value. Stable employment and an increase in added value during the observed period is also the reason for the increase in productivity in the food industry (13%). In agriculture, a simultaneous decrease in employment (-14%) and an increase in added value (8%) contributed to a moderate increase in productivity (26%). A greater increase in value added than the increase in employment in forestry contributed to the 7% productivity growth. In the chemical industry and pharmacy, productivity growth in the period 2008-2017 remains fairly static (1.5% growth). The only industry that is characterized by a decline in productivity in the period under consideration is the production of electricity.

### Catalytic role of Research, development and innovation (RDI) sector and commercial enabling institutions in bioeconomy development

In Slovenia, a vibrant Research, development and innovation (RDI) sector is operating, engaging in state-of-the art applied research and technology development in various bioeconomy-related fields of science ( Bartol *et al.*, 2014). This sector, consisting of both, public research institutions and private companies, can play a stronger catalytic role in unlocking the bioeconomy potentials as it is currently the case. In some sectors, which can be regarded as the cornerstones of the national manufacturing sector ( *e.g.*, pharmaceutical industry), RDI is strongly integrated with the industry. In other sectors, these linkages are less pronounced, or even not adequately established. The industry is reluctant to act as the sole investor in new technologies for different reasons ( *e.g.*, focus on cost efficiency, demand-side risks, lacking financial leverage), while the technology developers also seek for returns that surpass the capacities that are not attainable at the usual scale of enterprises operating in (conventional, or new) bioeconomy-related manufacturing sectors. To some extent, this gap has been successfully tackled within industry-research partnerships, developed within the national Smart Specialisation Strategy ( Cohen, 2019).

Slovenia has a vigorous network of enabling institutions supporting innovative and development-oriented entrepreneurial projects. Technology parks and business incubators provide professional business support services, such as favourable lease of business premises and start-up mentoring support. Business accelerators offer professional consultation and seed financing for innovative start-ups. Both programs are complemented with public funding. Market for venture capital is less developed, limited mainly to specialised products of banks and insurance companies. All the above described services are general, not relating specifically to bioeconomy ( Juvančič *et al.*, 2021a).

## System governance

### The institutional context of bioeconomy in Slovenia

Slovenia belongs to the (ever smaller) group of EU Member States that have not yet made commitments in terms of the preparation of a dedicated national bioeconomy strategy. No tangible progress can be reported with the respect with the long-term strategic orientation of bioeconomy among the national strategic priorities in Slovenia. It needs to be accentuated however that bioeconomy-related priorities are defined in a number of strategic documents and policy initiatives. Among national strategic documents, the following can be pointed out: Slovenian Sustainable Smart Specialisation Strategy 2021–27 ( MGRT, 2022), Slovenian Development Strategy 2030 ( Šooš, 2017), Slovenian Industrial Strategy 2030 ( MGRT, 2021), Comprehensive National Energy and Climate Plan 2030 ( Celoviti nacionalni energetski in podnebni načrt Republike Slovenije, 2020), and National Development Strategy of Agriculture and Food System ( MKGP, 2021). Bioeconomy is also strongly represented in the major inter-ministerial initiative, called Comprehensive Strategic Project for Decarbonising Slovenia.

The status of bioeconomy in Slovenia is elusive also in the institutional sense. There is no ministry or other government body that can be described as an institutional holder of the bioeconomy portfolio. This comes as no surprise as bioeconomy is a relatively new and cross-sectoral policy concept that spans over a number of (conventional) policy areas. The institutional status of bioeconomy appears to be challenging, particularly in governance systems, which prefer clear sectoral boundaries. What is more important though, is the fact that inter-ministerial coordination on various issues related with bioeconomy development is operating. It includes several portfolios, among them most prominently the following ones: (i) Environment and spatial planning, (ii) Agriculture, forestry and food, (iii) Economy and technology development, (iv) Science and education, and (v) European Affairs. No clear leadership can be identified in this coordination. Rather, the leadership/coordination role of institutions changes from one case to another. While this coordination has not yet converged towards a dedicated national bioeconomy strategy, the coordination is largely lacking also at the level of policy instruments and measures ( *e.g.*, criteria for selection of operations, coverage of related investments from different funds).

### Instruments and measures supporting bioeconomy development in Slovenia

As outlined above, contents related to bioeconomy development are only indirectly covered in national strategies and programming documents. As a result, even at the implementation level of these documents, as a rule, we do not find instruments and measures that would explicitly support the development and commercial adoption of biobased innovations, nor the further development and growth of bio-based value chains. Rather than this, investments, RDI actions and other activities introducing circular bioeconomy principles are increasingly emerging in some generic policy instruments, such as:

a) targeted support for micro- and SMEs in the early stages of commercialization;
b) pilot projects under the national Resilience and Recovery Plan;
c) projects aimed at the economic restructuring of fossil-based industries within the Just Transition Fund;
d) sectoral structural measures ( *e.g.*, investment support, training), aimed at the improvement of environmental sustainability, valorisation of by-products, or closing the (material/energy) loops;
e) support for RDI and transfer of (bio-based) technological innovations in industrial production ( *e.g.*, Common Agricultural Policy’s European Innovation Projects (EIP));
f) systemic measures to promote the transition to sustainable and circular solutions.

### Setting up the strategy: need for context-based solutions

The idealized model of circular bioeconomy is based on continuous and cost-effective access to industrially relevant quantities of biomass of homogeneous composition, its gradual decomposition in large integrated biorefineries into simpler (chemical, material) building blocks, which are then integrated a wide range of biobased products. The process is following the cascading use principles—starting with high value-added products and finishing with the energy use. Economic entities interact in the development of new technologies and processes (bioeconomic clusters) and in the exchange of material and energy flows (industrial symbiosis). The transition towards circular bioeconomy and its growth depends also on the wider supporting environment. It consists of a business supporting system supporting the early-stage companies, capable venture capital market to meet the firms' growth potentials, and the state with stable business environment, responsive legal framework, and consistent policy support.

In reality, the utilization of the development potential of the bioeconomy is context-based. The development of circular business models in the context of the Slovenian bioeconomy differs from the idealized model described above in practically all elements. It starts with a small-scale and fragmented production structure in primary sectors with low labour productivity. Similar holds for the conventional manufacturing sectors, which mainly operate at the SME scale. The potential for adding value of residues and by-products in production, processing and consumption remains largely untapped. In the absence of biorefinery capacities, the interest of industrial actors to expand biobased value chains, develop circular technological solutions and business models, remains rather low. The latter is present both on the side of industry, and on the side of public development policies.

The design of circular business models suitable for the conditions of the Slovenian bioeconomy needs to seek for innovative solutions adapted to this context.

## Overview of internal and external factors affecting the development of bioeconomy in Slovenia

The preceding two sections provide a structured description of the components, institutions and drivers that form the system of the national bioeconomy. Broadly speaking, two sets of information can be distinguished: the first one relates to the resource base, sectoral structure, innovation transfer and economic performance of bioeconomy, while the second one is concerned with the system governance. As the first step in setting the strategy, a conventional approach is applied by synthesizing and arranging the key findings into (internal) Strengths and Weaknesses, and (external) Opportunities and Threats (SWOT).

The SWOT elements are presented in separate tables and grouped along the following factors affecting bioeconomy:

- Biomass supply (BS), which describes the primary production of (agricultural, forest-based and aquatic) biomass, their interaction with related ecosystem services , as well as the infrastructural and logistic considerations;
- Bioeconomy value chain (BVC), which describes the sectoral mix of bioeconomy, degree of inter-sectoral linkages and multiplier effects, the level of (technological and organisational) sophistication, as well as the status of enabling environment ( *i.e.*, financial capital and other types of business support);
- RDI, which assesses the quality of bioeconomy-related research and development its integration in the development and transfer of industrial innovations;
- Institutional and policy framework (IPF), which outlines the status of bioeconomy among the strategic development priorities of the national economy, its institutional setup and integration into policy planning, types, scope and mutual coordination of supporting instruments;
- Competitive bioeconomy products (CBP), which outline the demand trends for biobased technologies and components in technology-intensive sectors, which accelerate (horizontal, vertical) integration along the extended bioeconomy value chains.

We start with the description of Strengths, which can be seen as the key internal factors shaping the growth of the bioeconomy in Slovenia ( Table 1).

**Table 1.** Strengths of the bioeconomy in Slovenia.

Code *	Description
S-BS1	Diverse and sustainably managed resources in agricultural and forestry production, associated with several ecosystem services, untapped potential for valorisation.
S-BS2	High percentage of forest areas (58% of country area) and the consequent high production potential of Slovenian forests.
S-BS3	Under-exploited flows of residual biomass from (diversified) primary agricultural production and by-products of food production.
S-BS4	Bioeconomy strongly represented in the manufacturing sector (conventional and novel bioeconomy sectors account for 28% of GVA at factor costs)
S-BVC1	Strong manufacturing industry and growing (demand-driven) interest of manufacturing firms to move towards bio-based technologies and to close energy and material loops.
S-BVC2	Vibrant enabling environment for supporting start-ups, growing number of bioeconomy-related spin-offs based on knowledge generated in (also public) RDI institutions.
S-RDI1	Internationally renowned applied research in various technologies of advanced bioeconomy capable of delivering hands-on solutions to industrial clients.
S-RDI2	Established development networks and strategic partnerships linking RDI with the economy and development policy for transitioning into a circular bioeconomy.
S-RDI3	Participation of public research organisations in the projects of the leading European platforms and programmes, transferring good practices from the EU to the national level.
S-RDI4	Stable system of financing for applied research projects based on guidelines garnered from stakeholder initiatives on priority RDI topics.
S-RDI5	Growing SME participation in Horizon 2020, EU networks and bioeconomy related RDI.
S-IPF1	Strong strategic commitments for systemic transition to a circular, regenerative, low-carbon economy in Slovenia, inter- ministerial coordination and international coaching.
S-IPF2	Strong policy commitment towards bioeconomy development, reflected in particular in the national Smart specialization strategy.
S-IPF3	Public support for networks and partnerships linking RDI with the industry.
S-IPF4	Green public procurement system with its direct and indirect impacts on the demand for biobased solutions ( *e.g.*, Incorporation of biobased construction materials).
S-CBP1	Growing number of firms, developers and early adopters of innovation, with a presence in international markets and providing a good practice to others.

* BS (Biomass supply); BVC (Bioeconomy value chain); RDI (Research, development and innovation); IPF (Institutional and policy framework); CBP (Competitive bioeconomy products); SME (Small and medium-sized enterprise)

In setting the strategy for the future development of the bioeconomy in Slovenia, its Weaknesses need to be taken into account as well. They are outlined in Table 2.

**Table 2.** Weaknesses of the bioeconomy in Slovenia.

Code *	Description
W-BS1	Technology lag and productivity gap in primary bioeconomy sectors, in particular in agriculture.
W-BS1	Limited ability to secure industrially relevant quantities of biomass due to fragmented production structure in primary sectors; lack of cooperation—horizontally and vertically.
W-BS2	Industrially relevant quantities, but low value-added of forest-wood biomass (export of roundwood, use of roundwood for energy purposes).
W-BS3	Well organized monitoring of waste streams, but very limited or no systemic monitoring of by-products and side-streams of biomass in manufacturing sectors, consequently reduced potential for the cascading use of biomass side-streams.
W-BVC1	Limited potential for developing scalable biobased value chains due to small-scale and fragmented plants for biomass processing.
W-BVC2	Low level of business integration in 'conventional' bioeconomy-related industries, making it harder to develop industrial-scale biorefineries, or leverage for the development of bioeconomy clusters.
W-BVC3	Successful businesses in various bioeconomy sectors but operating as individual firms on (usually niche) markets, lacking the capability, or willingness, to integrate into local/regional value chains.
W-BVC4	Weak financial leverage of companies in both conventional and 'new' bioeconomy sectors to make (investment-intensive and commercially risky) shifts to bio-based materials and technologies.
W-BVC5	Limited leverage of industrial and portfolio investors ( *e.g.*, venture capital), weak interest of financial service providers for higher-risk investments.
W-RDI1	Focus on public research institutions dedicated to basic research, rather than on new product and prototype development and demonstration.
W-RDI2	Insufficient feedback from the economy on RDI needs and applicability of results.
W-RDI3	Weak R&D infrastructure at the transition from laboratory to demonstration level (TRL 3-6) slows down innovation.
W-IPF1	No dedicated bioeconomy development strategy at the national level, leading to no systematic public support environment for the development of the bioeconomy.
W-IPF2	Low level of policy coordination, leading to scattered and often non-coordinated instruments and measures targeting particular sectors/aspects of bioeconomy.
W-IPF3	Lacking perception of bioeconomy as a strategic sector in public RDI funding and consequently inappropriate policies and lack of long-term funding.
W-IPF4	Fragmentation of resources in the R&D sector due to national funding being directed to small projects and groups; weak cooperation and integration of R&D.
W-IPF5	Administrative procedures and regulations inhibit development and commercialization through lengthy and uncertain implementation procedures.
W-CBP1	Difficulties faced by innovating companies to attract investments of sufficient critical mass as they are of moderate size and weakly integrated into regional clusters.

* BS (Biomass supply); BVC (Bioeconomy value chain); RDI (Research, development and innovation); IPF (Institutional and policy framework); CBP (Competitive bioeconomy products)

External trends that can positively affect the development, and performance of the bioeconomy in Slovenia are outlined in the Opportunities table ( Table 3).

**Table 3.** Opportunities for the bioeconomy in Slovenia.

Code *	Description
O-BS1	Increased provision of forest-wood biomass—partly due to improved utilization of the reserves in the annual wood increment.
O-BVC1	Development of a robust enabling environment, providing investment, ensuring scale-up, reducing risk and enabling a faster transition to market.
O-BVC2	Adopting national strategic commitments to improve knowledge-intensity in bioeconomy sectors ( *e.g.*, development departments, clusters, networks).
O-BVC3	Macro-regional cooperation and business integration to make better use of the bioeconomy potential ( *e.g.*, BIOEAST).
O-BVC4	Closing local/regional loops of biomass use by setting up a network of small-scale modular biorefineries for the processing of different biomass sources.
O-RDI1	Technological and management know-how resulting from in the wood and paper industries.
O-RDI3	Better use of opportunities provided by the European Research Area.
O-RDI4	Internationalization and participation of stakeholders in RDI strategic partnerships.
O-IPF1	Wider economic and social context (need for reduction of fossil resources and more efficient use of biomass by-products / waste streams).
O-IPF2	Growing awareness on the need for legislative changes towards environmental sustainability.
O-IPF3	The integration of bioeconomy content covered by the country's strategic development documents.
O-IPF4	Post-pandemic recovery developing the circular bioeconomy through appropriate investment, planning and inter-sectoral coordination.
O-CBP1	Increasing long-term demand for biobased technologies and products (due to positive consumer perception and improving price-cost relationship of biobased products).
O-CBP2	Growing demand for bio-based products in some important export sectors of the Slovenian manufacturing sectors.

* BS (Biomass supply); BVC (Bioeconomy value chain); RDI (Research, development and innovation); IPF (Institutional and policy framework); CBP (Competitive bioeconomy products)

Future development of the bioeconomy in Slovenia needs to take into account also the limiting external factors. They are outlined in Table 4.

**Table 4.** Threats for the bioeconomy in Slovenia.

Code *	Description
T-BS1	(Un)availability of biomass (forest and agricultural) due to the impact of climate change.
T-BS2	Potential conflicts between alternative uses of biomass and the risk of over-exploitation of renewable carbon sources.
T-BVC1	Capital-intensive and technologically advanced competition for the purchase of (mainly woody) biomass in neighbouring regions.
T-BVC2	The high capital cost of setting up efficient industrial operations for cascading use of biomass.
T-BVC3	Large investments in biorefinery capacity (demonstration development and industrial) in the wider EU region and limited access to new value chains.
T-BVC4	Inadequately sited biomass processing plants can disturb the price equilibrium, especially in the case of inappropriate subsidy policies ( *e.g.*, feed-in tariffs for bioenergy).
T-RDI1	Lack of appropriate definition of priority areas and objectives of bioeconomy development may result in continued sporadic RDI work on individual projects.
T-RDI2	Loss of development and investment momentum at the transition to higher TRLs due to financial, technical, organisational challenges.
T-RDI3	Technology closedness: Many biomass processing technologies are protected by long-standing patents.
T-IPF1	Neglect of the bioeconomy in planning for post-pandemic recovery, reorientation of focus in public policies ( *e.g.*, national security policy).
T-IPF1	Sporadic rather than systemic progress due to an uncoordinated legislative framework.
T-CBP1	Negative public opinion, linked in particular to the energy use of biomass and negative experience with poorly designed support policies in the past.
T-CBP2	Unfavourable price-cost ratios of bio-based materials and technological solutions.
T-CBP3	Reduced public confidence in bioeconomy-related innovations: 'greenwashing' or promoting projects with a doubtful (environmental, material, economic) results.

* BS (Biomass supply); BVC (Bioeconomy value chain); RDI (Research, development and innovation); IPF (Institutional and policy framework); CBP (Competitive bioeconomy products)

## Strategic propositions as derived from the SWOT analysis

### Biomass supply

In order to address the opportunities associated with favourable long-term trends on the demand-side (O-CBP1, O-CBP2), actions are needed to overcome the technology lag and productivity gap in the primary sectors of the bioeconomy, particularly in agriculture (W-BS1), and to better valorise the associated ecosystem services (S-BS1). Due to the small-scale and fragmented structural conditions associated with agricultural and forestry production (W-BS2, W-BVC1), further actions are needed in terms of the integration of primary producers into producer organizations. This would not only strengthen their bargaining position in the value chain but would bring potentials for improved logistical flows for residual biomass (S-BS3) *e.g.*, by storage, or partial processing of biomass to improve the cost efficiency of transport, and its durability.

Mobilisation of industrially relevant quantities of biomass to address the growing demand (O-CBP1, O-CBP2) is particularly relevant in the forestry sector with the relatively high and growing (S-BS2), but poorly utilized production potential (W-BS3). The changing species composition of forests and faster growth (O-BS1) associated with climate change (T-BS1) offer an opportunity for bioeconomy growth, although this will require a long-term reorientation of the associated manufacturing sectors (growth opportunities especially for the novel bioeconomy sectors). This would be needed also in order to improve the sectors’ position in the competition with the capital-intensive and technologically advanced competition in neighbouring regions (T-BVC2).

For a better mobilization of biomass side-streams, improvements are also needed in the monitoring system (W-BS4), which would enable better data availability about the available quantities and technologically relevant characteristics of biomass for further processing along the cascading principles.

### Bioeconomy value chain

Unlocking bioeconomy potentials along the bioeconomy value chains in Slovenia (O-CBP1, O-CBP2) should take place in two directions. Similarly, to the primary bioeconomy sectors, the reserves of the conventional manufacturing sectors of bioeconomy in Slovenia (food production, wood processing) lie in boosting the sectors' productivity and value added (S-BS4), partly also in the closing the material and energy loops within their operations. The second trajectory is more demand-driven. Its forerunners are firms, which are integrated into international value chains (S-BVC1) and include some of the key national manufacturing ( *e.g.*, chemical, automotive, electrical) and other sectors ( *e.g.*, construction), where the demands and needs for the transition to bio-based materials and technological solutions is increasing (S-CBP1). Increased demand for biobased final products from these sectors (O-CBP2) creates opportunities for growth along its upstream (technology developers) and downstream (primary and conventional manufacturing) sectors.

In order to unlock the potentials for a more integrated and sustainable bioeconomy in Slovenia, improvements are needed in the mobilization and utilization side-streams and residues of agricultural and forest-wood biomass. This will require overcoming the challenges of fragmented structure of the production and processing facilities in agri-food and forest-wood sectors (W-BS2).

Another challenge is a low level of horizontal and vertical integration along the bioeconomy value chains (W-BVC2, W-BVC3). This should not be misinterpreted by the general absence of technologically advanced and competitive firms in sectors operating along these chains. On the contrary, their number and significance is increasing (S-BVC1). What is lacking however is the low level of their integration, or at least cooperation. As a result, most of the firms in bioeconomy sectors are operating at the SME scale. Consequently, a large percentage of primary products in agriculture and forestry are valorised outside the national economy, and the conditions for biorefining of biomass side-streams at industrial scale is hardly attainable. Setting up a network of small-scale modular biorefineries (O-BVC4), combined with macro-regional cooperation and business integration (O-BVC3) would significantly improve the potentials for sustainable valorisation of biomass and economic performance (value added, employment) of the bioeconomy sectors within the national economy.

Better valorisation of biomass sidestreams through the installation of biorefining capacities and business integration will require development of a robust enabling environment, providing investment, ensuring scale-up, reducing risk and enabling a faster transition to market (O-BVC1). A vibrant generic business enabling environment (S-BVC2) provides a good groundwork, whereas improvements are needed in the financial leverage for (investment-intensive and commercially risky) innovative approaches towards biomass valorisation (W-BVC4, W-BVC5). Improvements are required also in terms of increased knowledge-intensity in bioeconomy sectors by strengthening industrial RDI through clusters and networks (O-BVC2). Internationalization and participation of industrial partners (O-RDI4) in international RDI strategic effort ( *e.g.*, through their participation in CBE JU projects) can accelerate innovation transfer. This can also control for the threat of being left out from the industrial innovation community (T-RDI3).

## Research, development and innovation (RDI)

The SWOT analysis points out a vibrant research RDI community engaged in applied life sciences and other relevant science disciplines for bioeconomy, able to deliver hands-on solutions to industrial clients (S-RDI1, S-RDI2). Research institutions and teams are well integrated into international RDI effort (S-RDI3). Investments in research and development and publications in this area are constantly increasing. The system of revolving (national) financing of research programmes (S-RDI4) brings the stability needed for a long-term applied research work. Increased involvement of end-users in research effort (S-RDI5) can also be seen as an asset on which to improve innovation adoption in the bioeconomy-related manufacturing sectors, which is currently assessed as weak (W-RDI3, W-RDI4). Another asset to improve the innovation adoption is also a vigorous start-up community (S-BVC2). Although these firms are operating at the micro-scale and in the early stages of the business cycle, they can be seen as the harbingers of the entrepreneurial transition to the bioeconomy.

Despite the obvious progress, there are still potentials for improvements in bringing research closer to the commercially viable outputs by new product and prototype development, or demonstration activities. To achieve this, refocusing of the operation is needed on both sides: (mostly public) research institutions should adopt applied research and innovation more ambitiously (W-RDI1), whereas the industry should also take a more proactive role in commissioning applied research and in providing feedback on the current research results (W-RDI2). Technological and management know-how resulting from the strong presence of the ‘conventional’ biobased industries in the structure of national manufacturing sector (S-BS4, O-RDI1) is an asset on which the cooperation of RDI and industrial partners can be strengthened. Additional funding of research excellence and international collaboration through the European Research area and dedicated public-private partnerships ( *e.g.*, CBE JU) would further stimulate these processes.

### Institutional and policy framework

Despite the proven long-term political commitment in Slovenia to the generic strategic development goals that comfortably accommodate the ambition of making better use of the bioeconomy potentials towards sustainable, more integrated and better performing bioeconomy (S-IPF1), institutional status and strategic significance of bioeconomy is unclear. This is reflected also in the fact that Slovenia is one of the seven EU Member States without a dedicated national bioeconomy strategy (W-IPF1).

Furthermore, review of national strategic documents and bioeconomy-related policies listed in the System governance section reveals that bioeconomy is not explicitly identified among the national strategic priorities in Slovenia. It needs to be accentuated though, that inter-ministerial coordination on various issues related with bioeconomy development is operating. Elements of (circular) bioeconomy have been integrated into various strategic documents and policy instruments (S-IPF1, S-IPF2). Bioeconomy-related themes are relatively strongly represented in the key strategy documents, such as Slovenian sustainable smart specialization strategy (S5), Slovenian industrial strategy 2030, Comprehensive national Energy and climate plan 2030, and the Comprehensive strategic project for the decarbonization of Slovenia.

At the level of the implementation of instruments and measures, the coordination between various ministry portfolios and funds is largely lacking (W-IPF2). From the point of view of final beneficiaries, especially in the case of large, integrated multi-sectoral projects, the lack of coordination (e.g., different criteria for selection of operations, different financial rules, overlaps of eligible operations between different funds on one side, uncovered areas of support on the other) may bring confusion and reduce the motivation of eligible beneficiaries to participate in supported actions.

For effective public support to a circular bioeconomy, it will be necessary to adjust and coordinate several government portfolios related to bioeconomy (i.e., environment, agriculture, industry and technology, research and innovation, education, employment). Setting at least a National bioeconomy Action plan would be a step in this direction.

Apart from the strategic planning, stronger inter-ministerial coordination is required in particular in the planning and implementation of supporting instruments and measures. Coordinated policy instruments ( *e.g.*, European Agricultural Fund for Rural Development (EARDF) and European Regional Development Fund (ERDF)) may stimulate intensified cooperation of firms along the value chain, bringing benefits both, in resource use sustainability, and in overall economic performance. Such coordination is required also to encourage the development of biorefinery capacities, which represent the key link in the formation of comprehensive multi-sectoral value chains.

Public policies can have an impact on the demand side of the market for biobased technological solutions and materials. The most straightforward tool for this is the system of public procurements, which—apart from the direct market effect with the institutional purchase—brings positive demonstration effects for private (corporate and individual) buyers. Positive experiences from the established system of Green public procurements in agri-food, energy and construction sectors (S-IPF4) can serve as an incentive to upgrade the instrument to other relevant biobased commodity markets ( *e.g.*, packaging materials, cleaning agents, stationery *etc.*).

An often overlooked aspect that has a strong impact on market introduction of biobased technologies and products, relates to the regulatory issues, such as product certification, standardisation and market authorisation procedures. The current situation in Slovenia is unfavourable. Lengthy and bureaucratically cumbersome regulatory procedures with uncertain outcomes are seen as a weakness (W-IPF5) that needs a serious overhaul.

Public policies can also provide effective indirect support for market mobilisation of biomass side-streams by establishing a monitoring system. Most likely this would require legal tightening in the direction of mandatory reporting. Although this is an unpopular measure, the long-term gains in terms of the availability of market information and consequently, commoditising and the establishment of market exchange with biomass side-streams and by-products would overcome the costs and initial dissatisfaction.

### Competitive bioeconomy products

For a serious qualitative leap towards (resilient, circular, sustainable) bioeconomy, all actors operating in the bioeconomy sectors or directing the development of bioeconomy in Slovenia, need to significantly strengthen their effort. This involves reaching a wide public consensus on the strategic importance and institutional consolidation of the bioeconomy.

Establishment of the National Bioeconomy Hub could be seen as a step in this direction. The hub would serve as a platform for mutual exchange of information, the dissemination and exchange of expertise, and the creation of business opportunities through cooperation. Industry associations and/or the chamber of commerce should be encouraged to draw up their own vision document on the bioeconomy. It makes sense to institutionalise a public-private partnership in the form of a bioeconomy hub or centre, linking knowledge institutions with industry, and to involve knowledge institutions and industry. Institutionally, it would be expedient to assign the role of a hub to an already operating platform with similar tasks. With the implementation of the Smart Specialization Strategy, the coordinating role is attributed to Strategic development innovation partnerships (SRIPs).

Another action to overcome the identified untapped potential for business integration (W-CBP1) is to identify national industrial leaders in bioeconomy and motivate them to act as integrators of regional bioeconomy value chains. Actions leading towards the support of their investment decisions ( *e.g.*, financial and equity input in the form of public-private partnerships) should be undertaken to motivate such actions.

## Ethics and consent

Ethical approval and consent were not required.

## Data Availability

No data are associated with this article.
