# Supplementary material for: Strategic concept paper for Bioeconomy in Slovenia: from a patchwork of good practices to an integrated, sustainable and robust bioeconomy system
Source: Open Res Eur. 2023 Oct 3;3:167. [Version 1] doi: 10.12688/openreseurope.16181.1 (PMC10665601; doi:10.12688/openreseurope.16181.1)
Supplement: Peer review history

# Reviewer report: Reviewer response for version 1

**DOI:** 10.21956/openreseurope.17468.r35948

**Published:** 2023-11-22

**Authors:** Matteo Vizzarri

**Affiliations:** 1 Università degli Studi di Milano, Milan, Italy

The authors made efforts to explore challenges and solutions for unlocking the potential for bioeconomy at national scale, in Slovenia. The article first develops throughout evaluating current state, challenges and potential by sub-sector, i.e. agriculture and forestry, manufacturing, research and development. Then, the authors introduce a possible framework for integrating bioeconomy-related solutions. Finally, a SWOT analysis – built on biomass supply, value chain, research, policy, and products – paves the way to key solutions for promoting / further developing bioeconomy in Slovenia.

 I have only minor comments:

- Key references to data sources are missing, particularly in the intro section. I would suggest to recall them with footnotes.
- Strategic propositions are to some extent described in isolation. The authors could provide some examples of potential synergies or side-effects when developing or implementing more than one solution simultaneously (e.g. biomass used in bioenergy vs. building construction)
- Message in Figure 1 is not fully clear to me. Do the authors want to show that Slovenian figures are much lower than EU27 average?  

To me, the article is of a broad interest for the CEE region, especially for sharing potential solutions to further develop bioeconomy paths.

Where applicable, are recommendations and next steps explained clearly for others to follow? (Please consider whether others in the research community would be able to implement guidelines or recommendations and/or constructively engage in the debate)

Partly

Does the article adequately reference differing views and opinions?

Yes

Are all factual statements correct, and are statements and arguments made adequately supported by citations?

Partly

Is the rationale for the Open Letter provided in sufficient detail? (Please consider whether existing challenges in the field are outlined clearly and whether the purpose of the letter is explained)

Yes

Is the Open Letter written in accessible language? (Please consider whether all subject-specific terms, concepts and abbreviations are explained)

Yes

Reviewer Expertise:

Sustainable forest management, forest ecosystem services, landscape planning, forest-based economy

I confirm that I have read this submission and believe that I have an appropriate level of expertise to confirm that it is of an acceptable scientific standard.

# Reviewer report: Reviewer response for version 1

**DOI:** 10.21956/openreseurope.17468.r35503

**Published:** 2023-10-30

**Authors:** Ants-Hannes Viira

**Affiliations:** 1 Institute of Agricultural and Environmental Sciences, Estonian University of Life Sciences, Tartu, Estonia; 2 Agricultural Research Department, The Centre of Estonian Rural Research and Knowledge, Jõgeva, Estonia

The paper addresses a relevant problem - bioeconomy development gap in the Central and Eastern European Member States of the EU, the case of Slovenia in specific. 

 The paper opens by describing the current situation of the bioeconomy related economic sectors in Slovenia, paying special attention to primary biomass production, conventional bioeconomy processing industry as well as research, development and innovation. The main challenges are explained - primary biomass export, lacking processing capacity, and fragmentation of the structure of enterprises. 

 Next, the paper reviews the current policies in place and strategies in Slovenia, that address the bioeconomy. Also here, the picture is fragmented. 

 Based on the SWOT analysis, the authors develop the main strategic activities that could be addressed in the comprehensive national bioeconomy strategy (which is currently missing). Here, the authors focus mainly on two aspects - what are the biomass resources available in Slovenia, and how to make better use of those resources through dedicated research, development and innovation. 

 Finally, the authors suggest to establish a national bioeconomy hub to create a platform for bioeconomy actors for information exchange, business development, research, development and innovation collaboration, as well as for elaborating further policy recommendations. 

 I think the paper has merit. Stakeholders from several Central and Eastern European Member States of the EU will recognize similar problems like the authors outline in the case of Slovenia. Thus, there are lessons learned for wider set of countries. Also, I would like to thank the authors for formulating 'idealized model of circular bioeconomy'. This is very well summarized and useful explanation for everybody who is interested in the subject.

 I have the following suggestions for the authors, which are mainly addressing some issues about the clarity of presentation:

1. The authors refer to this paper as a 'concept paper'. However, it remains vague in what respect this paper is a 'concept paper'. If the 'concept paper' status is important for this case, this nuance could be explained.
2. In a couple of places in the paper, the authors use the term 'hybrid bioeconomy sectros'. As this term is not well known, perhaps the meaning of hybrid could be explained to readers.
3. The authors state that there is room for further harvesting of timber in Slovenia. Since the debates on carbon capture and storage by land use, land use change and forestry (LULUCF) sectors is ongoing in the EU, the authors could additionally discuss what could be the implications for Slovenian (and possibly other countries') forest based bioeconomy development.
4. In the list of strengths in Table 1, formulation on S-BS3 seams more like a weakness 'Under-exploited flows of residual biomass from (diversified) primary agricultural production and by-products of food production.' The authors could consider revision of wording or moving this factor to the weaknesses category.
5. In the list of opportinities (Table 3), several items are worded like activities to be done to develop Slovenian bioeconomy (e.g. Development of a robust enabling environment, providing investment, ensuring scale-up, reducing risk and enabling a faster transition to market.). To the knowledge of reviewer, the opportunitiers should refer to factors that are external to the analysed system. Therefore, I would suggest to consider rewording of the items in Table 3, so that they focus on opportunities eternal to bioeconomy sector. 

Where applicable, are recommendations and next steps explained clearly for others to follow? (Please consider whether others in the research community would be able to implement guidelines or recommendations and/or constructively engage in the debate)

Yes

Does the article adequately reference differing views and opinions?

Yes

Are all factual statements correct, and are statements and arguments made adequately supported by citations?

Yes

Is the rationale for the Open Letter provided in sufficient detail? (Please consider whether existing challenges in the field are outlined clearly and whether the purpose of the letter is explained)

Partly

Is the Open Letter written in accessible language? (Please consider whether all subject-specific terms, concepts and abbreviations are explained)

Yes

Reviewer Expertise:

Agricultural policy, agricultural development, agricultural economics, agri-food value chains, bioeconomy policy.

I confirm that I have read this submission and believe that I have an appropriate level of expertise to confirm that it is of an acceptable scientific standard.
